# cAMP/PKA Regulates Osteogenesis, Adipogenesis and Ratio of RANKL/OPG mRNA Expression in Mesenchymal Stem Cells by Suppressing Leptin

**DOI:** 10.1371/journal.pone.0001540

**Published:** 2008-02-06

**Authors:** Der-Chih Yang, Huey-Jen Tsay, Shan-Yang Lin, Shih-Hwa Chiou, Mei-Jane Li, Tai-Jay Chang, Shih-Chieh Hung

**Affiliations:** 1 Institute of Clinical Medicine, National Yang-Ming University, Taipei, Taiwan; 2 Institute of Pharmacology, National Yang-Ming University, Taipei, Taiwan; 3 Institute of Neuroscience, National Yang-Ming University, Taipei, Taiwan; 4 Department of Medical Research and Education, Veterans General Hospital - Taipei, National Yang-Ming University, Taipei, Taiwan; 5 Department of Orthopaedics and Traumatology, Veterans General Hospital - Taipei, National Yang-Ming University, Taipei, Taiwan; University of Birmingham, United Kingdom

## Abstract

**Background:**

Mesenchymal stem cells (MSCs) are a pluripotent cell type that can differentiate into adipocytes, osteoblasts and other cells. The reciprocal relationship between adipogenesis and osteogenesis was previously demonstrated; however, the mechanisms remain largely unknown.

**Methods and Findings:**

We report that activation of PKA by 3-isobutyl-1 methyl xanthine (IBMX) and forskolin enhances adipogenesis, the gene expression of PPARγ2 and LPL, and downregulates the gene expression of Runx2 and osteopontin, markers of osteogenesis. PKA activation also decreases the ratio of Receptor Activator of the NF-κB Ligand to Osteoprotegerin (RANKL/OPG) gene expression – the key factors of osteoclastogenesis. All these effects are mediated by the cAMP/PKA/CREB pathway by suppressing leptin, and may contribute to PKA stimulators-induced *in vivo* bone loss in developing zebrafish.

**Conclusions:**

Using MSCs, the center of a newly proposed bone metabolic unit, we identified cAMP/PKA signaling, one of the many signaling pathways that regulate bone homeostasis via controlling cyto-differentiation of MSCs and altering RANKL/OPG gene expression.

## Introduction

Bone remodeling has long been considered to be controlled through the bone multicellular or metabolic unit (BMU), which contains two cellular activities: osteoclastic bone resorption and osteoblastic bone formation. However, recent genetic studies have shown that there is no obligatory tight cross-control of bone formation and bone resorption *in vivo*. In osteopetrotic patients, the osteoclasts are either absent or non-functional, yet bone formation is unaltered [1]. The osteopenic transgenic mice, in which osteoblast ablation leads to a complete arrest of bone formation accompanied by bone loss, illustrate that the bone-resorption function is also independent of bone formation [2].

Several tissues constitute the skeletons, including bone, bone marrow, periosteum, perichondrium and cartilage. Therefore, it is possible that cells other than osteoblasts or osteoclasts are involved in bone remodeling. Mesenchymal stem cells (MSCs), first isolated and referred as mechanocytes or CFU-F cells by Friedenstein [3], can be derived from the bone marrow [4], trabecular bone [5] or dental root [6], have the capacity of self-renewal and can differentiate into several distinct cell types, including osteoblasts and adipocytes. The differentiation of MSCs into either osteoblasts or adipocytes is exclusively and transcriptionally regulated by two key transcription factors, Runx2 (also called Cbfa1) and PPARγ, respectively [7]–[11]. An inverse relationship between the differentiation of adipocytes and osteoblasts was demonstrated in cell and animal models and was found in various human diseases [12]. For example, decreased bone formation is accompanied by an increase in bone marrow adipogenesis in age-, immobility- and corticosteroid-associated osteoporosis [13]–[15]. However the signaling pathways and molecular mechanisms that regulate this still remain largely unknown [16].

Cyclic 3′,5′-adenosine monophosphate (cAMP) is a key intracellular signaling molecule, whose main function is to activate the cAMP-dependent protein kinase A (PKA). PKA is a heterotetrameric holoenzyme containing a regulatory (R) subunit dimer and two catalytic (C) subunits. PKA is activated when four molecules of cAMP bind to the R subunit dimer, two to each R subunit, releasing two free active C subunits responsible for phosphorylation of key substrates [17]. The cAMP/PKA signaling pathway critical in phenotypic specification and transition in the adult and developing central nervous systems (CNS) has also been demonstrated to induce neuronal differentiation in a number of primary or cloned neural precursor cells derived from the CNS [18], [19], and induce neuronal differentiation and neurite outgrowth in MSCs [20]. An elevated plasma and urinary cAMP has long been found in osteoporotic patients [21], however, it is unclear where cAMP/PKA signaling plays an important role in controlling bone homeostasis and regulating cyto-differentiation of MSCs. In an attempt to investigate this issue in the current study, we indeed found that the addition of stimulators of cAMP/PKA signaling in a defined adipocyte induction medium (AIM) enhanced MSC adipogenesis and also found that cAMP/PKA signaling inhibited MSC osteogenesis. Thus, the current study provides a possible molecular mechanism for the reciprocal relationship between adipogenesis and osteogenesis, a well known differentiation phenotype.

## Methods

### Cells

Primary MSCs were isolated from iliac crest of donors with informed consents and grown in DMEM-LG (Invitrogen, Grand Island, NY) supplemented with 10% fetal bovine serum (FBS, Invitrogen), 100 U/ml penicillin, 10 Ag/ml streptomycin, and 0.25 µg/ml amphotericin B at 37°C under 5% CO2 atmosphere. The medium was changed twice a week and a subculture was performed at 1∶3 to 1∶5 split every week. The human MSC strain has been described previously and was originally derived from the bone marrow of a 61-year-old female donor. This strain has been transfected with a plasmid containing the type 16 human papilloma virus proteins E6/E7 [22]. This strain is now referred to as KP-MSCs and grown in the same culture condition with primary MSCs. Using flow cytometry, KP-MSCs express CD29 (α1 integrin), CD44 (hyaluronan receptor), CD90 (Thy-1), CD105 (endoglin), SH2, and SH3 (MSC markers). KP-MSC cells were harvested and used at passages 28–32.

### Cytodifferentiation and cell treatments

One day before the initiation of differentiation, cells were seeded in growth medium at a density of 10,000 cells/cm^2^. For adipogenic induction, the cells were washed twice with PBS (Phosphate buffered saline) and treated in a defined Adipogenic Induction Medium [AIM: DMEM-LG supplemented with 10% FBS, 50 µg/ml ascorbate-2 phosphate (Sigma, St. Louis, MO), 10^−7^ M dexamethasone (Sigma), 50 µg/ml indomethacin (Sigma), and 10 µg/ml insulin (Sigma)]. For experiments investigating cAMP/PKA modulators on adipogenic differentiation, cells were treated in AIM with the addition of 0.45 mM 3-isobutyl-1-methylxanthine (IBMX), (Calbiochem, La Jolla, CA), 100 µM forskolin (Calbiochem), 11 µM Sp-cAMP (Calbiochem), 20 µM PKI (Sigma), or 11 µM Sp-cAMP+20 µM PKI. Medium was changed every 3 days. After the appearance of morphologic features of differentiation, cells were used for histochemical study.

### Histochemical study

Cells were washed twice with PBS, fixed in 10% formalin for over 1 hour at room temperature, and stained with Oil Red-O (Sigma) to show adipogenic differentiation for 2 hours. The Oil Red-O working solution was prepared by mixing 15 ml of a stock (0.5% in isopropanol) and 10 ml of distilled water and filtering through a PDVF membrane (0.2 µm) filter. Quantification of lipid accumulation was achieved by extracting Oil Red-O from stained cells with isopropanol and measuring the OD of the extract at 510 nm using an ELISA reader (Sepctra MAX 250; Molecular Devices, Sunnyvale, CA).

### Measurement of leptin secretion

Leptin secretion was measured using commercially available ELISA kits according to the manufacturer's instructions (BioSource International, Camarillo, CA) to determine the concentration of leptin in the conditioned medium of cells cultured in complete growth medium or in AIM with or without the PKA modulators.

### Reverse transcription-polymerase chain reaction (RT-PCR)

Total RNA was extracted using the Trizol reagent (Invitrogen) according to the manufacturer's specifications. First strand cDNA synthesis was performed using Superscript III reverse transcriptase (Invitrogen), Random primer (Invitrogen), 10 mM dNTPs (Invitrogen), 5× First Strand synthesis buffer, 0.1 M DTT, and RNaseOUT ribonuclease RNase inhibitor (Invitrogen). PCR was performed using cDNA as the template in a 50 µl reaction mixture containing a specific primer pair of each cDNA according to the published sequences (Table 1). Each cycle consisted of the following three steps: denaturation for 45 sec at 94°C, annealing for 1 min at 51–58°C, and 90 sec of elongation at 72°C. The reaction products were resolved by electrophoresis on a 1.5% agarose gel and visualized with ethidium bromide.

**Table 1:** PCR primers

Gene	Sequence (Sense and antisense)	Product size (bP)
β-actin	5′-GCACTCTTCCAGCCTTCCTTCC-3′	515
	5′-TCACCTTCACCGTTCCAGTTTTT-3′	
PPARγ2	5′-CCTATTGACCCAGAAAGCGATTC-3′	595
	5′-GCATTATGAGACATCCCCACTGC-3′	
LPL	5′-TGTAGATTCGCCCAGTTTCAGC-3′	490
	5′-AAGTCAGAGCCAAAAGAAGCAGC-3′	
Runx2	5′-GTTTGTTCTCTGACCGCCTC-3′	318
	5′-CCAGTTCTGAAGCACCTGA-3′	
Osteopontin	5′-CTAGGCATCACCTGTGCCATACC-3′	373/331
	5′-CAGTGACCAGTTCATCAGATTCATC-3′	
Leptin	5′-TCTTGTGGCTTTGGCCCTATCT-3′	181
	5′-CCAGTGTCTGGTCCATCTTGGATA-3′	
RANKL	5′-AATAGAATATCAGAAGATGGCACTC-3′	486
	5′-TAAGGAGGGGTTGGAGACCTCG-3′	
OPG	5′-GCTAACCTCACCTTCGAG-3′	324
	5′-TGATTGGACCTGGTTACC-3′	

### Real-Time RT PCR

Expressions of the target gene PPARγ2, Runx2, RANKL, OPG and the endogenous reference GAPDH were quantified using primers, probes, and standards. The primers and TaqMan probes were designed using the software Primer Express (Applied Biosystems, Warrington, UK). RT-PCR was performed according to a TaqMan two-step method using an ABI PRISM 7700 sequence detection system (Applied Biosystems). The non-template controls, standard dilutions, and tumor samples were assayed. Total RNA isolated from MSCs treated in various conditions was reversely transcribed to cDNA using an oligo deoxythymidine primer. A 20 µl volume of PCR reaction mixture was used containing 50 ng of the sample cDNA: 9 µl; 20× Target Assay Mix or 20× Endogenous Control Assay Mix: 1 µl; 2× TaqMan Universal Master Mix (with or without AmpErase UNG): 10 µl (Applied Biosystems). The PCR cycling conditions included an initial phase of 2 min at 50°C, followed by 10 min at 95°C for AmpErase, 45 cycles of 15 min at 95°C and 1 min at 60°C. Real-time amplification of the genes for PPAR *γ*2 (product number Hs00234592_m1), Runx2 (product number Hs00231692_m1), RANKL (product number Hs00243519_m1), OPG (product number Hs00171068_m1), and GAPDH (product number Hs99999905_m1) was performed using the ABI Assays on Demand primers© and Taqman® universal PCR master mix on the ABI 7500 real-time PCR machine according to the manufacturer's instructions (Applied Biosystems). Analysis of the results was carried out using the software supplied with the machine. The software calculates PPAR γ2, Runx2, RANKL and OPG expression relative to the GAPDH housekeeper gene (delta CT) and then relative to controls (delta delta CT) using the fluorescence threshold of the amplification reaction and the comparative CT method.

### Western blot analysis

Protein was extracted from subconfluent cell cultures. The cells (8×10^5^) were rinsed with PBS and lysed in 0.2 ml of protein extraction reagent (M-PER, Pierce, Rockford, Illinois) plus protease inhibitor cocktail (Halt™, Pierce) for 5 min on ice. Protein concentrations were determined using the BCA assay (Pierce). After being heated for 5 min at 95°C in a sample buffer, equal aliquots of the cell lysates were run on a 10% SDS polyacrylamide gel. Proteins were transferred to PVDF membrane filters. The filter was blocked for 1 hour with TBS containing 5% nonfat dry milk and 0.05% Tween 20 and then incubated overnight at 4°C with the primary antibodies. The filter was washed 3 times for 10 minutes each with TBS containing 0.05% Tween 20. Bound primary antibodies were detected by incubating for 1 hour with horseradish peroxidase-conjugated goat anti-mouse or anti-rabbit IgG (BD PharMingen, San Diego, CA). The filter was washed and developed using a chemiluminescence assay (Perkin Elmer Life Science, Inc., Boston, MA). Densitometric analysis was carried out using Image Master 2D Elite software (Amersham Biosciences). Activation of CREB was detected by Western blot analysis of the phosphorylation state using phospho-CREB antibodies (Cell Signaling Technology). The level of CREB phosphorylation was calculated as the density ratio of bands corresponding to pCREB and CREB.

### Protein kinase A catalytic activity assays

cAMP-dependent PKA activities were assayed using the PepTag Assay for nonradioactive detection of PKA (Promega, Madison, WI) on the basis of the phosphorylation of fluorescent-tagged PKA-specific peptides. The cell lysate for PKA protein was prepared using a hypotonic extraction buffer (20 mM Tris, pH 7.5, 5 mM EDTA, 1 mM PMSF, and 10 mg/ml aprotinin). Aliquots of the PKA preparation and PKA catalytic subunit as a positive control were incubated for 30 min at 30°C in PepTag PKA 5× reaction buffer (100 mM Tris-HCl, pH 7.4, 50 mM MgCl2, and 5 mM ATP) and 0.4 µg/µl of the PKA-specific peptide substrate PepTag A1 (L–R–R–A–S–L–G; Kemptide). The reaction was stopped by heating for 10 min at 95°C. Phosphorylation of the PKA-specific substrates was used to measure kinase activity. Phosphorylated and unphosphorylated PepTag peptides were separated on a 0.8% PH 8.0 Tris-HCl agarose gel by electrophoresis. For the PKA assays, 10 µg of protein was applied.

### DNA delivery methods

For transfection of cells with pmaxGFP or pcDNA3 or pcDNA3-CREBR287L, Nucleofector technology (AMAXA Biosystems, Cologne, Germany) was used with each nucleofection sample containing 4 µg of DNA, 4×10^5^ cells, and 100 µl of Human MSC Nucleofector Solution. The transfection was carried out under the program C-17 of the Nucleofector device, as recommended by the manufacturer. The transfected cells were then suspended in an appropriate volume of 20% FBS supplemented DMEM-LG medium and seeded for further culture. After 24 hours of incubation, the medium was replaced by AIM with 100 µM Forskolin for 3 days. The efficiency of transfection as evaluated by the expression of EGFP in cells transfected with pmaxGFP vector was more than 50%.

### Zebrafish maintenance and histological staining

Zebrafish embryos were maintained in Embryo Medium at 28°C. To visualize developing bone, the embryos were immobilized in ice water, then fixed for 24 hours in buffered 4% paraformaldehyde. After washing twice in PBS, they were dehydrated in 50% and 100% ethanol each for 24 hours. They were placed in 0.05 mg/ml Alcian Blue in 3∶7 glacial acetic acid : 100% ethanol for 20 min. Alcian Blue-stained zebrafish were then cleared in 1% KOH for one hour. The fish were then transferred to 2 mg/ml Alizarin Red in 1% KOH for 1 hour and cleared in 20% glycerol in 1% KOH for 40 min. Alizarin Red-stained zebrafish were photo-pictured or preserved in 50% and then 80% glycerol in 95% ethanol, followed by 100% glycerol.

### Raman microspectroscopic study

Each specimen for spectroscopic study was dried at 25°C, 50% relative humidity (RH) for one week, and then immediately measured. The biophysical characteristics and molecular compositions of the excised samples were determined by using a dispersive micro-Raman spectrophotometer (Ventuno, Jasco Co., Tokyo, Japan) equipped with a 30 mW green (532 nm) solid-state laser as standard. The pixel resolution was 1.3 cm^−1^. The spectra were obtained in the 400–4000 cm^−1^ range [23].

### Vibrational spectroscopic study

The calcified sample was dried for one week at 25°C, 50% RH condition, and then used for spectral investigation to determine its biophysical characteristics and molecular compositions by using both Fourier transform infrared (FTIR) microspectroscopy (Micro FTIR-200, Jasco Co.) with transmission technique and confocal micro-Raman spectrophotometer (Ventuno, Jasco Co., Tokyo, Japan) equipped with a 30 mW green (532 nm) solid-state laser as standard via non-destructive analysis [23].

## Results

### PKA stimulators enhance adipogenesis, inhibit osteogenesis and decrease the ratio of RANKL/OPG gene expression

**Figure 1:**
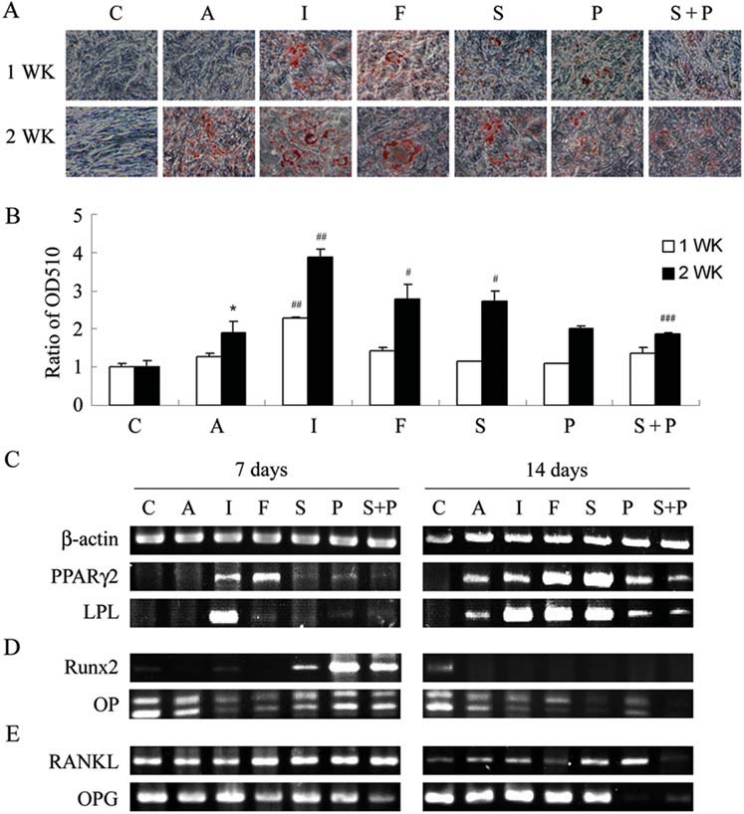
PKA modulators influence adipogenesis, gene expression for adipogenesis, osteogenesis and osteoclast-inducing factors in KP-MSCs. (A) Enhancement of adipogenesis by IBMX, forskolin and Sp-cAMP. Cells were treated in complete growth medium [C] or in adipocyte induction medium without [A] or with the indicated modulator of PKA activity [I: IBMX (0.45 mM), F: forskolin (100 µM), S: Sp-cAMP (11 µM), P: PKI (20 µM), S+P: Sp-cAMP+PKI] for 7 and 14 days. Differentiated adipocytes were detected by the accumulation of lipids, which were stained with Oil Red O. (B) Oil Red O staining as OD510. The results displayed in the bar graphs are quantitative analytic data from three independent experiments and are presented as the meanSD values. Each ratio was normalized to the control, and significance was determined by Student's t-test. (* p<0.05 versus C; ^#^ p<0.05, ^# #^<0.01 versus A, ^# # #^ p<0.05 versus S). RT-PCR analysis of gene expression of makers for adipogenesis (C), osteogenesis (D), and osteoclast-inducing factors (E) after treatment with PKA modulators.

**Figure 2:**
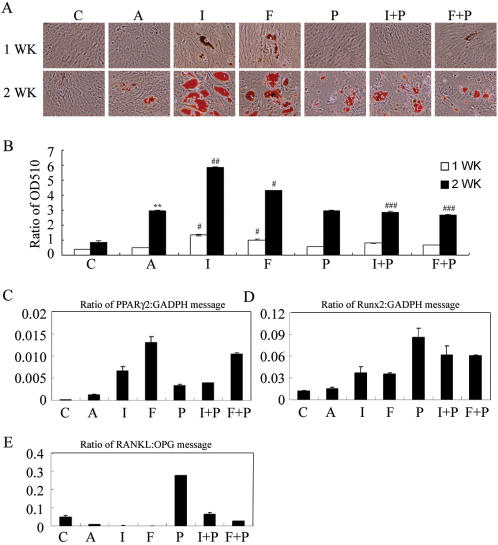
PKA modulators influence adipogenesis, gene expression for adipogenesis, osteogenesis and osteoclast-inducing factors in primary MSCs. (A) Enhancement of adipogenesis by IBMX and forskolin. Cells were treated in complete growth medium [C] or in adipocyte induction medium without [A] or with the indicated modulator of PKA activity [I: IBMX (0.45 mM), F: forskolin (100 µM), P: PKI (20 µM), I+P: IBMX+PKI, F+P: forskolin+PKI] for 7 and 14 days. Differentiated adipocytes were detected by the accumulation of lipids, which were stained with Oil Red O. (B) Oil Red O staining as OD510. The results displayed in the bar graphs are quantitative analytic data from three independent experiments and are presented as the meanSD values. Each ratio was normalized to the control, and significance was determined by Student's t-test. (** p<0.01 versus C; ^#^ p<0.05, ^# #^<0.01 versus A, ^# # #^ p<0.05 versus I or F). Real-Time RT-PCR analysis of the gene expression ratio of PPARγ2/GADPH (C), Runx2/GADPH (D), and RANKL/OPG (E) after treatment with PKA modulators for 7 days.

In a first attempt to identify the optimal conditions for adipogenic differentiation, we found that the addition of 3-isobutyl-1 methyl xanthine (IBMX) significantly increased adipogenic differentiation by primary MSCs (Figure S1). Because IBMX is a phosphodiesterase inhibitor which prevents cAMP degradation to activate PKA, the cAMP/PKA pathway is hypothesized to stimulate adipogenesis. The primary MSCs have limited life span with an average number of population doublings of 38 and tend to change their differentiation potential during in vitro expansion [24]. Furthermore, the usual methods that are used for transfection are not easily applied in primary MSCs. Therefore, a previously developed KP-MSC line and primary MSCs were used in the current study to extend the stimulatory effect of IBMX on adipogenesis [22]. To investigate the role of the cAMP/PKA signaling pathway in MSC differentiation, we first compared the effects of several modulators of the cAMP/PKA pathway, including stimulators such as IBMX, forskolin, and Sp-cAMP, inhibitors such as PKI and combinations of both on the adipogenesis of MSCs by measuring Oil Red-O staining (Fig. 1A and 2A). Both KP- and primary MSCs treated in AIM began to initiate adipogenesis, and the extension of treatment for 2 weeks significantly increased the intensity of Oil Red-O staining (Fig. 1A and 2A). However, incubation of KP-MSCs in a complete growth medium did not induce any increase in Oil Red-O staining, suggesting that the cells did not undergo spontaneous differentiation into adipocytes. Interestingly, IBMX added in AIM further increased Oil Red-O staining both at 7 and 14 days (Fig. 1B and 2B). The substitution of IBMX with either forskolin or Sp-cAMP significantly increased Oil Red-O staining at day 14 in comparison with the AIM group. The addition of PKI in AIM did not cause any significant change in Oil Red-O staining, whereas, PKI significantly inhibited the stimulatory effect of IBMX , forskolin or Sp-cAMP on Oil Red-O staining as compared with the IBMX , forskolin or Sp-cAMP group at 14 days (Fig. 1B and 2B). Meanwhile, the effect of IBMX, forskolin or Sp-cAMP on lipid vesicle appearance was also inhibited by the addition of PKI (data not shown).

The effects of PKA modulators on KP-MSC adipogenesis was further investigated by RT-PCR to determine the mRNA expression of the key transcription factor, PPARγ2, and a lineage marker of adipocytes, LPL (Fig. 1C). PPARγ2 and LPL were not detected from cells in complete growth medium at 7 and 14 days or in AIM at day 7, but they could be detected in AIM at day 14, further confirming the initiation of adipogenesis in AIM but not in the complete growth medium. When adding IBMX or other PKA stimulators such as forskolin and Sp-cAMP in AIM, the expression of PPARγ2 and LPL mRNA was up-regulated either at 7 or 14 days. Adding PKI in AIM did not result in any significant change in mRNA expression of PPARγ2 and LPL, whereas, PKI decreased the stimulator effect of Sp-cAMP on mRNA expression of these two genes. Levels of mRNA expression analyzed by real-time RT-PCR in primary MSCs also revealed the same results. Treatment with IBMX and forskolin significantly induced the expression of PPARγ2 and the addition of PKI at the same time blocked the inductive effects (Fig. 2C). These results all together suggest a stimulatory role of cAMP/PKA signaling in MSC adipocyte differentiation and a low stimulation of cAMP/PKA signaling in cells treated with AIM.

An inverse relationship between MSC adipogenesis and osteogenesis was well known, therefore, we further investigated the effect of cAMP/PKA signaling pathway on gene expression of Runx2, the earliest transcription factor of osteoblast differentiation [7], [8], and one osteoblast lineage markers, osteopontin (OP) (Fig. 1C). The level of Runx2 expression was low in cells treated in complete growth medium, and increased after the extension of the culture period for 14 days. This data support the previous finding that MSCs underwent spontaneous differentiation into osteoblasts when the cells reached confluence. The level of Runx2 expression did not significantly increase in cells treated in AIM with or without any PKA stimulators. However, a substantial increase in Runx2 expression was observed in cells treated with PKI, suggesting the stimulatory effect of PKA inhibitor on MSC osteogenesis (Fig. 1D). Furthermore, cells treated in AIM without or with the PKA modulators for 14 days, which all expressed PPARγ2, did not express any gene expression of Runx2, indicating the inverse relationship between adipogenesis and osteogenesis by MSCs. Cells in growth medium expressed OP and alkaline phosphatase (data not shown) gene expression, suggesting these genes are not exclusively expressed by osteoblasts. However, the gene expression of OP was down-regulated in cells treated in AIM with the addition of a PKA stimulator, including IBMX, forskolin and Sp-cAMP at 7 or 14 days as compared with the expression in AIM (Fig. 1D). Expression of Runx2 mRNA was also significantly increased in primary MSCs when treated with PKI (Fig. 2D). These results all together suggest an inhibitory role of cAMP/PKA signaling in MSC osteoblast differentiation.

Receptor Activators of NF-κB Ligand (RANKL) and osteoprotegerin (OPG, a decoy receptor for RANKL) coordinate with one another to play a critical role in the regulation of osteoclast formation, and the relative presence of RANKL and OPG dictates osteoclastogenesis [25], [26]. PKA stimulators such as IBMX, forskolin and Sp-cAMP, which stimulated the expression of PPARγ2, inhibited the expression of RANKL (day 7 versus 14), stimulated the expression of OPG (day 7 versus 14), and thus decreased the RANKL/OPG ratio (Fig. 1E). On the other hand, inhibitors of PKA activity, which stimulated the expression of Runx2, inhibited the expression of OPG (Sp-cAMP+PKI versus PKI in day 7 and day 14), and thereby increased the RANKL/OPG ratio (Fig. 1E). Consistent with the effect in KP-MSCs, PKI also significantly increased the ratio of RANKL/OPG gene expression of primary MSCs when treated in AIM with or without PKA stimulators (Fig. 2E). These data suggest that osteoblast differentiation in MSCs, which is inhibited by cAMP/PKA signaling, is associated with an increase in the RANKL/OPG gene expression ratio.

### PKA stimulators stimulate adipogenesis, inhibit osteogenesis and increase osteoclast-inducing potential via suppressing leptin expression and secretion

**Figure 3:**
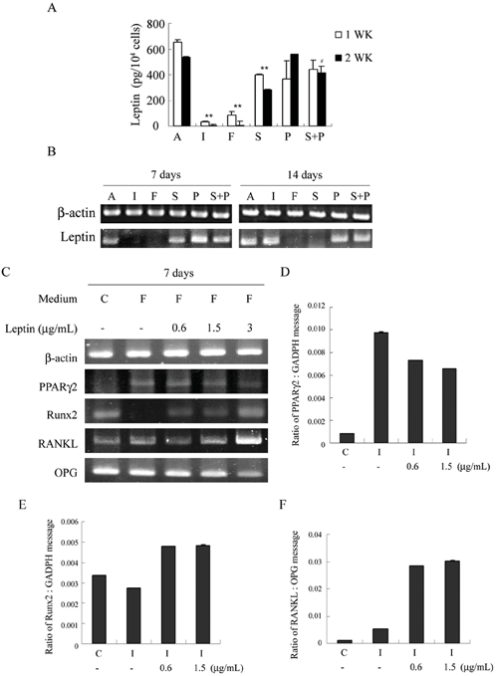
PKA stimulators regulate gene expression for adipogenesis, osteogenesis and osteoclast-inducing factors by suppressing leptin. (A,B) Suppression of leptin secretion and transcription by IBMX, forskolin and Sp-cAMP. Cell treatments were the same as Fig. 1. (A) Leptin concentrations in the conditioned medium of the last 2 days were measured by ELISA and normalized by cell numbers. The figure shows representative data from two independent experiments with three replicates for each condition; data are presented as the meanSD values, and significance was determined by Student's t-test. (**<0.01 versus A; ^#^<0.05 versus S) (B) RT-PCR analysis of mRNA for leptin and β-actin. (C) Exogenous leptin inhibits forskolin-induced effects on gene expression of PPARγ2 and Runx2 and the RANKL/OPG gene expression ratio. Cells were cultured in complete growth medium [C] or treated in AIM with 100 µM forskolin [F] in the absence or presence of leptin (0.6, 1.5, 3 µg/ml) for 7 days. RT-PCR analysis for β-actin, PPARγ2, Runx2, RANKL and OPG. Real-Time RT-PCR analysis of the gene expression ratio of PPARγ2/GADPH (D), Runx2/GADPH (E), and RANKL/OPG. (F). Exogenous leptin inhibits the effects of AIM with IBMX (I: AIM with 0.45 mM IBMX) on gene expression of PPARγ2, Runx2 and the RANKL/OPG gene expression ratio.

Leptin is highly expressed and secreted by adipocytes residing in bone marrow and fat tissues [27]. The leptin secretion from adipocytes, however, is stimulated by insulin, one component of AIM [28], [29], and is also regulated by subtracts modulating PKA activity [30]. We, therefore, investigated the role of leptin in PKA stimulators-mediated stimulation of adipogenesis-dependent gene expression, inhibition of osteogenesis-dependent gene expression, and decrease in the ratio of RANKL/OPG gene expression in MSCs. First, we measured leptin levels in cells treated in AIM with or without PKA modulators. The baseline for leptin concentration in a conditioned medium (CM) from cells treated in AIM was around 600 pg/10^4^ cells both at 1 and 2 weeks. Treatment with PKA stimulators such as IBMX, forskolin and Sp-cAMP significantly inhibited leptin secretion, although there is less inhibition with Sp-cAMP (Fig. 3A). However, PKA inhibitors caused a significant increase in leptin secretion only at 2 weeks, when comparing the treatments with Sp-cAMP and with Sp-cAMP+PKI (Fig. 3A). We, then, studied the expression of mRNA encoding for leptin as a possible explanation for the decrease in leptin secretion. At first, we found that MSCs expressed mRNA for leptin receptor (OB-R), and neither incubation in AIM nor treatment with PKA stimulators or inhibitors caused any significant change. The baseline for leptin mRNA (OB) in AIM was detectable, and the treatment with PKA stimulators greatly decreased leptin gene expression (Fig. 3B). Coincident with the effect in inhibiting leptin secretion, Sp-cAMP inhibits leptin gene expression to a lesser degree. Interestingly, there was a great increase in leptin gene expression at 2 weeks of cells treated in AIM with IBMX, even though the leptin secretion in these cells was still far below baseline. However, the removal of IBMX from the medium in these cells induced leptin secretion (data not shown). These results are compatible with previous reports that leptin was secreted by adipocytes and suggest IBMX has a direct effect in inhibiting leptin expression and secretion.

To address whether PKA stimulators-mediated leptin suppression may be required for their regulation of MSC differentiation, namely induction of adipogenesis and inhibition of osteogenesis, we investigate whether the effects of PKA stimulators would be blocked by adding leptin exogenously. This was then demonstrated by the addition of exogenous leptin in cells treated in AIM with forskolin, which inhibited PPARγ2 expression, stimulated Runx2 expression and increased the ratio of RANKL/OPG in a dose-dependent manner (Fig. 3C). The effects of exogenous leptin on regulating adipogenic, osteogenic and osteoclast-inducing factor gene expressions were also demonstrated in cells treated in AIM with IBMX when evaluated by real time PCR (Fig. 3D–F). These data clearly answer that PKA stimulators induce adipogenesis, inhibit osteogenesis and decrease the RANKL/OPG gene expression ratio via suppressing leptin expression and secretion.

### PKA stimulators increase phosphorylation of CREB to regulate expression of leptin, PPARγ and Runx2 and the ratio of RANKL/OPG

**Figure 4:**
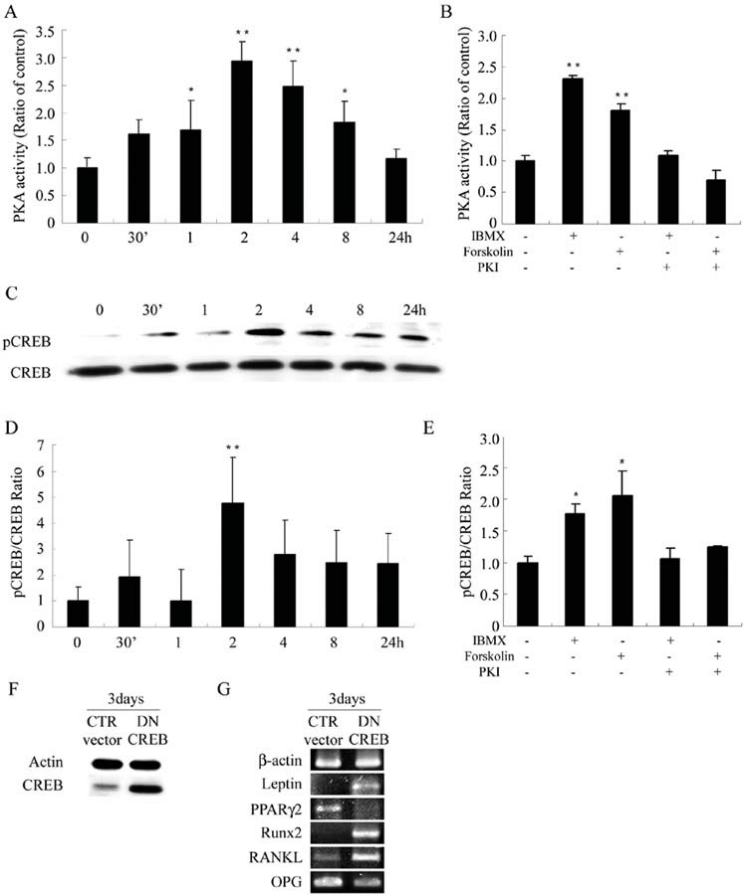
PKA stimulators increase phosphorylation of CREB to regulate gene expression of leptin, PPARγ2 and Runx2 and the RANKL/OPG ratio. Cells were treated in AIM with IBMX (0.45 mM) after serum starvation for 48 hours. (A) PKA activity increased immediately and reached the peak at 2 hours and gradually returned to the baseline at 24 hours. (B) Cells were treated in AIM with different combinations of IBMX (0.45 mM), forskolin (100 µM), and PKI (20 µM), and PKA activities were measured at 2 hours after treatment. (C,D) The same experiment of (A). Level of CREB phosphorylation was indicated as ratio of pCREB/CREB protein level. (E) The same experiment of (B) Levels of CREB phosphorylation were measured at 2 hours after treatment. Data are presented as the mean±SD values of three separate experiments, and significance was determined by Student's t-test. (* p<0.05, ** p<0.01 versus the baseline). (F,G) Suppression of forskolin-induced modulation of gene expression of leptin, PPARγ2, Runx2, RANKL and OPG by transfection with DN CREB. Cells were transfected with control (pcDNA3) or DN CREB vector for 48 hours, followed by treatment in AIM with forskolin (100 µM) for 3 days. (F) Western blot analysis of CREB protein in the transfected cells. (G) RT-PCR analysis of gene expression in transfected cells. Transfection with DN CREB abrogated forskolin-induced block of leptin expression, initiation of PPARγ2 expression, and inhibition of Runx2 expression, and increased the ratio of RANKL/OPG gene expression.

To determine whether PKA stimulators activate PKA in MSCs, cells were treated in AIM with IBMX, and PKA activity was assayed in serum-free conditions. Cells treated by serum depletion for 48 hours expressed a baseline of PKA activity, and the level of PKA activation increased immediately, reaching its peak, about threefold of baseline, at 2 hours after induction and gradually returned to the baseline at 24 hours after induction (Fig.4A). In a separate experiment with PKA activity analyzed at 2 hours after induction, while cells in IBMX or forskolin-containing AIM still expressed about twofold PKA activity, addition of the PKA inhibitor, PKI, blocked the activation of PKA by IBMX or forskolin (Fig. 4B). The CREB-binding protein transcriptional coactivator is an important regulator of gene expression through its ability to interact and cooperate with several transcription factors, such as CREB in response to cAMP/PKA signaling. Like its effect on modulating PKA activities in an induction medium, IBMX in AIM also phosphorylated CREB. CREB phosphorylation reached a peak level, more than fourfold the baseline, at 2 hours after induction, and maintained a twofold expression of the baseline level after 24 hours of induction (Fig. 4C and 4D). Similar to the block of PKA activity by PKI, phosphorylation of CREB by IBMX or forskolin was also blocked by the addition of PKI (Fig. 4E).

To directly assess the participation of CREB in directing differentiation into adipocytes or osteoblasts and controlling the ratio of RANKL/OPG, a control or a dominant-negative (DN) CREB plasmid, pcDNA3-CREBR287L, was transfected into MSCs before the initiation of differentiation (Fig. 4F). When compared with the control vector, transfection with DN CREB stimulated leptin expression, decreased PPARγ2 expression, increased Runx2 expression, and increased the ratio of RANKL/OPG gene expression (Fig. 4G). These results demonstrate that cAMP/PKA/CREB pathway regulates cyto-differentiation and osteoclast-inducing potential of MSCs.

### Leptin blocks PKA stimulator-induced bone loss in developing zebrafish

**Figure 5:**
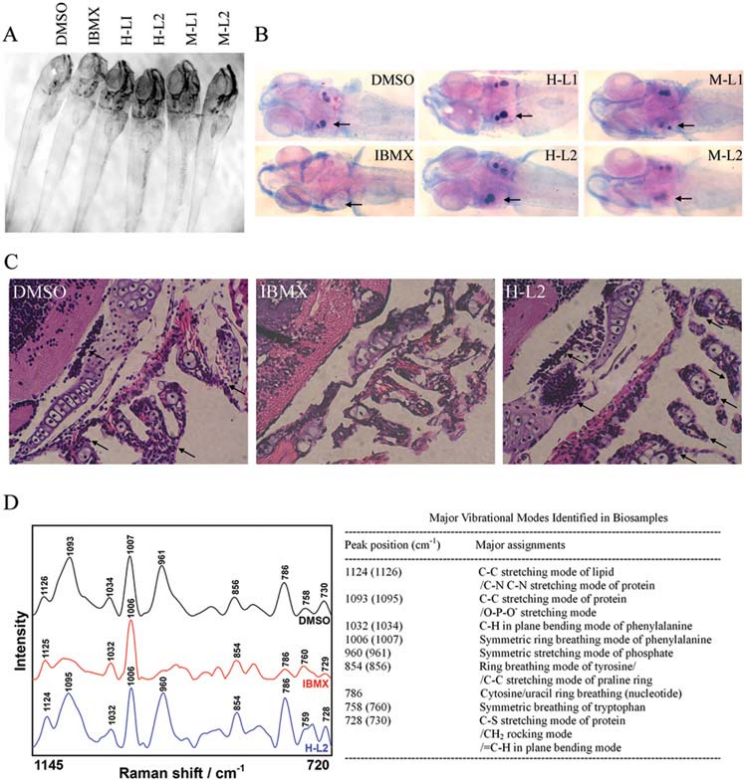
Leptin abrogates IBMX-induced lack of skeletal ossification in developing zebrafish. Embryos were treated in vehicle DMSO, IBMX (0.045 mM), and IBMX with human [H] or mouse [M] leptin (L1: 0.6 µg/mL, L2: 1.5 µg/mL) at 2 dpf. (A) Morphology of embryos at 8 dpf. (B) Alizarin Red S and alcian blue staining of whole zebrafish reveals normal skeletal ossification in DMSO-treated embryos at otolith (arrow), while absent skeletal ossification is evident in IBMX-treated embryos. The addition of human and mouse leptin both in low and high concentrations abrogates IBMX-induced absence of skeletal ossification. (C) Hematoxylin-and-eosin staining of zebrafish sections reveals impaired bone development in IBMX-treated embryos. Arrows indicate bone development with cell aggregation at otolith and pharyngeal arches. (D) Raman microspectroscopic analysis of bone development at otolith in zebrafish.

To investigate the *in vivo* role of activation of cAMP/PKA signaling on bone development, we used a zebrafish vertebrate model system. Since bone development is progressively increased at the beginning of larval stage embryos, IBMX treatment was initiated at 2 dpf (days after fertilization) [31]. The IBMX-treated embryos survived and had normal morphology for up to 8 dpf (Fig. 5A). Bone development was visualized in whole embryos by Alizarin Red S staining (Fig. 5B), along with hematoxylin-and-eosin staining of thin sections (Fig. 5C). In normal and vehicle (DMSO)-treated control embryos, mineralization as indicated by positive Alizarin Red S staining was apparent at the otoliths, and extensive skeletal development was evident in the cranial and pharyngeal region at 8 dpf (normal, n = 10; control, n = 8). No bone formation was observed in any of the IBMX-treated embryos at 8 dpf (n = 19). Although, leptin was identified in fish using an antibody against mouse leptin, the fish leptin is still not isolated [32]. Therefore, we use leptin both from human and mouse to investigate leptin effects on zebrafish bone formation. Interestingly, human and mouse leptin block IBMX-induced bone loss both at 0.6 and 1.5 µg/mL (H-L1, n = 14; H-L2, n = 15; M-L1, n = 17; M-L2, n = 14). These results were further evidenced by microscopic Raman spectroscopy (Fig. 5D). All of the embryos displayed a similar peak at 1007 cm^−1^ that is assigned to phenylalanine and frequently maintains unchanged at different samples. A unique feature in the Raman spectrum of IBMX-treated embryos was a lack of the large peak at 961 cm^−1^ and 1095 cm^−1^ corresponding to the ν_1_ symmetric stretching mode of the phosphate group of hydroxyapatite and the C-C stretching mode of protein/phosphate stretching mode of DNA and RNA, respectively (Fig. 5D). The former is an indication of bone formation, the latter is a predominant signal of DNA/RNA formation [33]. The value of peak intensity ratio of 961 cm^−1^/1007 cm^−1^, as a biomarker to investigate the extent of bone formation, was 0.82, 0.15 and 0.91 for DMSO, IBMX and IBMX with leptin, respectively. The similar value of 1095 cm^−1^/1007 cm^−1^ for DMSO and IBMX with leptin suggests that both samples had the same phosphate group in the backbone conformations of RNA and DNA after embryo development, as compared with the smaller value of 1095 cm^−1^/1007 cm^−1^ for IBMX. These findings confirm a critical role for leptin involvement in cAMP/PKA signaling-mediated *in vivo* bone loss in developing zebrafish. We were unable to investigate the role of leptin in adipogenesis in these embryos, because adipocytes have not been described in teleosts, and in other vertebrate species, fat deposition does not occur until the postnatal period.

## Discussion

The cAMP/PKA pathway has been demonstrated in 3T3-L1 cells, murine preadipocytes, to stimulate adipogenesis by activating the transcription factor CREB [34], [35]. However the direct effects of cAMP/PKA on the differentiation of MSCs are not well studied. In the current report, we explored the direct involvement of cAMP/PKA signaling in regulating MSC differentiation and its ability to control osteoclastogenesis. The addition of PKA stimulators in AIM enhanced Oil Red-O staining and the expression of markers for adipocytes (PPARγ2 and LPL), inhibited the expression of markers for osteoblasts (Runx2 and Op), and increased the RANKL/OPG ratio at the mRNA level. We further demonstrated that PKA stimulators repressed leptin synthesis and secretion in MSCs and that the effects of PKA stimulators on MSCs were blocked by the administration of exogenous leptin. These data suggest that PKA stimulators exerted their effects on MSCs via the repression of endogenous leptin. Furthermore, the direct participation of CREB in the effects of PKA stimulators was demonstrated through the use of a DN CREB plasmid, which blocked the effects of the PKA stimulator when transfected into cells before differentiation. These results suggest that cAMP-dependent PKA activity is sufficient to increase CREB activity and to regulate three distinct lineage differentiations (adipogenesis, osteogenesis and osteoclastogenesis) that are involved in bone homeostasis.

However, the effects of IBMX, forskolin and Sp-cAMP on levels of gene expression were inconsistent in some situations, such as the mRNA level of Runx2 after stimulation with Sp-cAMP for 7 days. To understand the underlying mechanism, the abilities of these three reagents to activate PKA have been compared with those of IBMX and forskolin stronger than that of Sp-cAMP (data not shown), which may be used to explain how cells treated with Sp-cAMP still expressed Runx2.

*In vitro* studies established that leptin enhanced the osteogenesis of human stromal cells, increased mineralization of the extracellular matrix, but inhibited adipogenesis [36], [37]. Similar findings have been obtained with other cell models [38]. However, it is not clear from the animal studies whether leptin is a stimulator or an inhibitor of bone growth, as intracerebroventricular infusion of leptin affects bone mass and returns the high bone mass phenotype in ob/ob mice back to wild-type levels [39], whereas systemic administration has been reported to induce loss of bone marrow adipocytes and increase bone formation in ob/ob mice [27], [40], [41] and to reduce bone loss in ovariectomized rats [42] and in tail-suspended female rats [43]. In the current study, we found that the induction of adipogenesis in MSCs was associated with a decrease in leptin secretion and gene expression. The suppression of leptin expression is believed to play a role in the initiation of adipogenesis because the administration of exogenous leptin inhibited adipogenesis. The addition of exogenous leptin from 0.6 to 3 µg/mL not only inhibited the expression of PPARγ2, but also stimulated the expression of Runx2 and the ratio of RANKL/OPG gene expression in a dose-dependent manner, indicating that leptin functions as a molecule rheostat that modulates bone homeostasis. Similar in *in vitro* MSC cultures, the role of leptin on bone homeostasis was also demonstrated in bone development of the *in vivo* zebrafish model. Although plasma leptin levels were reported with diurnal variation and variant among individuals [44], a significant relationship was observed between plasma leptin concentration, and the bone mineral mass and density in healthy elderly men and women [45], and the presence of vertebral fractures in postmenopausal women [46]. These results further support our finding that cAMP/PKA signaling inhibited bone formation by suppressing leptin.

**Figure 6:**
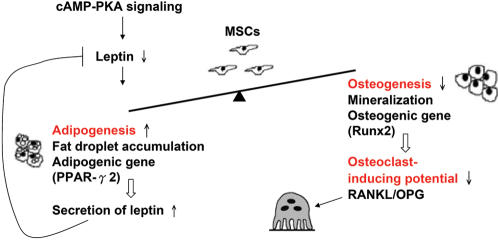
Proposed model of bone metabolic unit with the mesenchymal stem cell in the center. cAMP/PKA, one of the signaling pathways of bone homeostasis, regulating cyto-differentiation and osteoclast-inducing potential of MSCs by suppressing leptin.

Bone formation has been studied over the past 20 years by investigating the balance between osteoblast and osteoclast function based on the concept that bone homeostasis is controlled by these two cells. In the current paper, we propose a new bone metabolic unit which highlights the role of MSCs, and upon which we identified cAMP/PKA signaling, one of the many signaling pathways that regulate bone homeostasis, which might determine the constitution of the cellular components via directing adipogenesis, osteogenesis and osteoclastogenesis by controlling the release of leptin from MSCs (Fig. 6). This new concept has been supported by a study on MSC differentiation, which has shown that a 14-3-3-binding protein, TAZ, co-activates Runx2-dependent gene transcription while repressing PPARγ2-dependent gene transcription [16]. The concept is further supported by a most recent study by Zhao et al. [47]. They wisely identified 53 endogenous candidate suppressors of osteogenic specification by screening a synthetic SiRNA library targeting 5,000 human genes, which when silenced could initiate differentiation of MSCs into osteoblasts. 11 of those selected SiRNAs inhibited adipogenic differentiation of MSCs under conditions that induce adipogenic differentiation. Furthermore, cAMP was also identified to play opposing roles in osteogenesis vs. adipogenesis. The results from this and other studies show that the MSCs may be used to elucidate molecular signaling on the regulation of adult bone homeostasis by investigating the potential to differentiate into adipocytes and osteoblasts and to regulate osteoclastogenesis by changing the ratio of RANKL/OPG gene expression.

## Supporting Information

Figure S1 The adipogenic differentiation of primary MSCs as demonstrated by Oil Red O staining. Primary MSCs were induced in AIM with (A) or without (B) 0.45 mM IBMX for 3 weeks.
(1.85 MB TIF)
