# Supplementary figures and images for: cAMP/PKA Regulates Osteogenesis, Adipogenesis and Ratio of RANKL/OPG mRNA Expression in Mesenchymal Stem Cells by Suppressing Leptin
Source: PLoS One. 2008 Feb 6;3(2):e1540. doi: 10.1371/journal.pone.0001540 (PMC2212109; doi:10.1371/journal.pone.0001540)

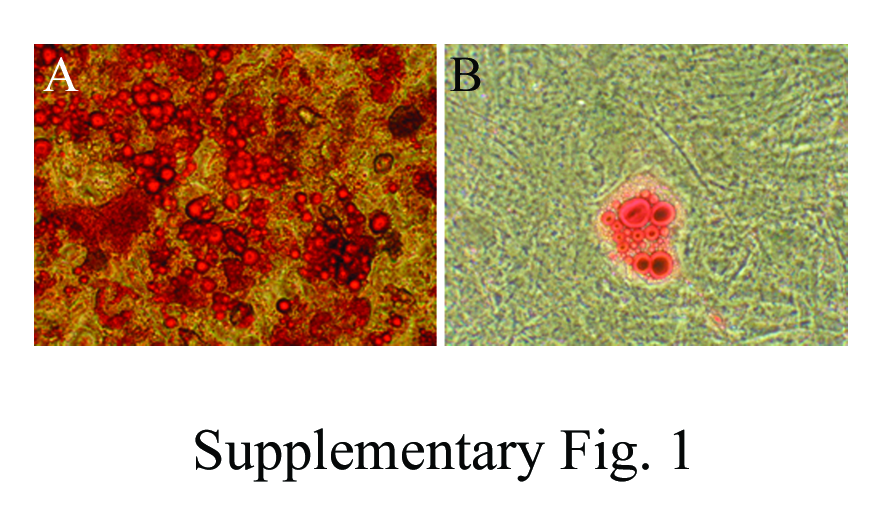

Supplement: Figure S1 — The adipogenic differentiation of primary MSCs as demonstrated by Oil Red O staining. Primary MSCs were induced in AIM with (A) or without (B) 0.45 mM IBMX for 3 weeks. (1.85 MB TIF) [file pone.0001540.s001.tif]
